# Can we bridge the gap? Knowledge and practices related to Diabetes Mellitus among general practitioners in a developing country: A cross sectional study

**DOI:** 10.1186/1447-056X-10-15

**Published:** 2011-11-24

**Authors:** Prasad Katulanda, Godwin R Constantine, Muditha I Weerakkody, Yashasvi S Perera, Mahesh G Jayawardena, Preethi Wijegoonawardena, David R Matthews, Mohamed HR Sheriff

**Affiliations:** 1 National Hospital of Sri Lanka, Colombo, Sri Lanka; Department of Clinical Medicine, Faculty of Medicine, Colombo, Sri Lanka; 2 Diabetes Research Unit, Faculty of Medicine, University of Colombo, Sri Lanka; 3 Department of Clinical Medicine, Faculty of Medicine, University of Colombo, Sri Lanka; 4 Ceylon College of General Practitioners, Colombo, Sri Lanka; 5 Oxford Centre for Diabetes, Endocrinology and Metabolism, University of Oxford, UK; 6 Post Graduate Institute of Medicine, Colombo, Sri Lanka

**Keywords:** Diabetes Mellitus, general practitioners, Sri Lanka, Primary care

## Abstract

**Background:**

Diabetes mellitus is becoming a serious public health problem in Sri Lanka and many other developing countries in the region. It is well known that effective management of diabetes reduces the incidence and progression of many diabetes related complications, thus it is important that General Practitioners (GPs) have sound knowledge and positive attitudes towards all aspects of its management. This study aims to assess knowledge, awareness and practices relating to management of Diabetes Mellitus among Sri Lankan GPs.

**Methods:**

A cross-sectional study was conducted among all 246 GPs registered with the Ceylon College of General Practitioners using a pre-validated self-administered questionnaire.

**Results:**

205 responded to the questionnaire(response rate 83.3%). Their mean duration of practice was 28.7 ± 11.2 years. On average, each GP had 27 ± 25 diabetic-patient consultations per-week. 96% managed diabetic patients and 24% invariably sought specialist opinion. 99.2% used blood glucose to diagnose diabetes but correct diagnostic cut-off values were known by only 48.8%. Appropriate use of HbA1c and urine microalbumin was known by 15.2% and 39.2% respectively. 84% used HbA1c to monitor glyceamic control, while 90.4% relied on fasting blood glucose to monitor glyceamic control. Knowledge on target control levels was poor.

Nearly 90% correctly selected the oral hypoglyceamic treatment for obese as well as thin type 2 diabetic patients. Knowledge on the management of diabetes in pregnancy was poor. Only 23.2% knew the correct threshold for starting lipid-lowering therapy. The concept of strict glycaemic control in preference to symptom control was appreciated only by 68%. The skills for comprehensive care in subjects with multiple risk factors were unsatisfactory.

**Conclusions:**

The study was done among experienced members of the only professional college dedicated to the specialty. However, we found that there is room for improvement in their knowledge and practices related to diabetes. We recommend continuing medical education and training programs to update GP's knowledge in order to improve health outcomes in this group of patients.

## Background

Diabetes mellitus is becoming a serious public health problem in Sri Lanka and many other developing countries in the region. Latest prevalence studies demonstrate a prevalence of 9.8% and 10.9% among adult males and females respectively [1]. These figures are expected to further increase resulting in escalating health care costs with primary care having to shoulder a larger burden in caring for these patients. At present majority of these patients are managed in the primary care, mostly by full-time General Practitioners (GPs).In Sri Lankan context any doctor having a medical degree (MBBS) and registered with the Sri Lanka Medical Council (SLMC) can practice as a GP. A general practitioner therefore plays a pivotal role in the management of diabetes mellitus in the community. The complexity and chronic nature of diabetes present many challenges to the family physician. With regard to diabetes the primary goal of these GPs would be to achieve and maintain optimal glycaemic control, prevent micro and macrovascular complications and thereby to improve patients' quality of life. It is well known that effective management of diabetes reduces the incidence and progression of many diabetes related complications [2-7]. Hence it is important that GPs have sound knowledge and positive attitudes towards all aspects of the management of this chronic disease including all the levels of prevention.

The American Diabetes Association (ADA), International Diabetes Federation (IDF) and many other organizations have developed evidence-based guidelines for the management of diabetes mellitus [8,9].As shown in table 1 there are specific diagnostic criteria for the proper diagnosis of diabetes for the patients to be properly identified and managed as well as to be cautious of over treatment. To improve the quality of care of patients with diabetes we need to evaluate the existing practice adopted by the GP's, who handles bulk of the diabetic patients at the community level. There is scarce information on awareness and attitudes of GPs in the management of diabetes at the primary care level. The present study was designed to fill this void in our knowledge using evidence-based guidelines of the American Diabetes Association (ADA) and International Diabetes Federation (IDF) as a bench mark [8,9]. The present study aims to determine the level of awareness, attitudes and practices related to diabetes mellitus in a group of GPs from Sri Lanka.

**Table 1:** American Diabetes Association diagnostic criteria for diabetes mellitus

1. HbA1c ≥ 6.5% *The test should be performed in a laboratory using a method that is NGSP certified and standardized to the DCCT assay□*
**OR**
2. FPG ≥ 126 mg/dL(7 mmol/L). *Fasting is defined as no caloric intake for at least eight hours.□*
**OR**
3. 2-h plasma glucose ≥ 200 mg/dL(11.1 mmol/L)during an OGTT. *The test should be performed as described by the World Health Organization, using a glucose load containing the equivalent of 75 g anhydrous glucose dissolved in water□*
**OR**
4. Random plasma glucose ≥ 200 mg/dL(11.1 mmol/L) *In a patient with classic symptoms of hyperglycemia or hyperglycaemic crisis *

□ In the absence of unequivocal hyperglycemia, criteria 1-3 should be confirmed by repeat testing.

## Methods

A cross-sectional study was conducted between October-December 2008 using a pre-tested self-administrated anonymous questionnaire. All 246 GPs registered with the Ceylon College of General Practitioners (CCGP) were invited to participate in the study. Since this is the only College dedicated to General Practice in Sri Lanka, we assumed that they would approximate with best practice in the country. The invitation to participate together with the questionnaire was mailed. The accompanying letter indicated that they should not refer books or journals or discuss with others in order answer the questions. After 2 weeks non-responders were sent reminders.

The questionnaire included sections on the diagnosis, glycaemic control, assessment and management of related risk factors (hypertension and dyslipidaemia), practices of screening for complications and delivery of vital health messages including life style modifications. In addition information regarding the experience of GPs, the volume of practice and proportion of diabetes consultations per week were collected. Case scenarios were also used to assess knowledge of GPs in management of a diabetic patients (e.g. A 35 year old patient with type 2 diabetes, who is 5 feet 6 inches in height, weighing 85 kg, blood pressure 140/100 mmHg, FBG 300 mg/dL, LDL cholesterol 200 mg/dL and smoking 10 cigarettes a day. He had a first degree family history of heart attacks in fifties. Physical examination was otherwise normal. The respondents were then required to choose answers for a set of questions that tested their decision-making in relation to the scenario.

The Diabetes Control and Complications Trial (DCCT), significantly changed the management principles of Diabetes mellitus from the 1990 s onwards [10]. DCCT study examined whether intensive treatment with the goal of maintaining blood glucose concentrations close to the normal range could decrease the frequency and severity of those complications. The DCCT provided quantifiable justification to healthcare providers that the additional expenses associated with intensive glycemic control and close monitoring of diabetes are cost effective. Similarly the UKPDS results confirmed and extended previous evidence supporting the hypothesis that hyperglycemia and its sequelae are a major cause of the microvascular complications of diabetes. This also indicated that the presence of hyperglycemia is a toxic state whether it occurs early or late in life and irrespective of its underlying cause[10,11]. It was assumed that the GPs knowledge should be updated for their clinical practice based on the importance of these two landmark studies. Thus the awareness of the trials among the GPs was also explored.

Ethical clearance for the study was obtained from the Ethics Review Committee of the Faculty of Medicine, University of Colombo. Data was double-entered, cross checked for consistency and analysed using SPSS version 14 (SPSS Inc., Chicago, IL, USA) statistical software package.

## Results

Two hundred and five (response rate 83.3%) responded to the questionnaire. The mean duration of practice after registration as a medical practitioner was 28.7 (SD ± 11.2) years. The average number of weekly consultations was 355 (SD ± 235) while those for diabetes related problems was 27 (SD ± 25) per week. Majority of GPs (96%, n = 196) manage diabetic patients by themselves with 24% seeking specialist opinion from time to time. Diabetic patient records were maintained by 182 (88.8%).

### Diagnostic testing

Urine sugar and HbA1c were used by 36.8% and 27.2% respectively as diagnostic tests in their practice (Table 2). However, 200 (99.2%) used fasting blood glucose for diagnosis, though only 48.8% knew the correct cut-off value for the diagnosis. In addition random blood sugar and oral glucose tolerance tests were also being carried out for diagnosis.

**Table 2:** Preferred management options by the GPs

Preferred diagnostic tests used for diagnosis of diabetes mellitus	
Fasting blood glucose	99.2%

Random blood glucose	54.4%

Oral glucose tolerance test	41.6%

Urine sugar	36.8%

HbA1c	27.2%

**Use of statins in patients with diabetes mellitus**	

With IHD	44%

When LDL>100 mg/dL	23.2%

In all diabetes	6.4%

When LDL>150 mg/dL	3.6%

When HDL >65 mg/dL	1.6%

**Use of low dose aspirin in patients with diabetes mellitus**	

With IHD	87.2%

With other risk factors	80.8%

All >40 years of age	32.8%

All diabetics	10.4%

Never	0.2%

**Knowledge on the cut off values for metabolic and blood pressure control**	

HbA1C	63.2%

Fasting Blood Sugar	43.2%

Blood pressure	43.2%

Triglycerides	31.2%

LDL Cholesterol	28.8%

### Monitoring glycaemic control

Fasting Plasma Glucose (FPG) was used by 90.4% for monitoring of glycemic control while 84.0% used HbA1c, 28.8% used urine sugar and 62.4% used Post Prandial Blood Sugar (PPBS).

### Screening for complications

Upon diagnosis of a patient most GPs routinely arrange for urine dipstick examination (75.2%), urine for microalbumin(88.0%), lipid profile (31.2%), serum creatinine (68.0%), and Electrocardiograms (80.0%) as initial investigations. A majority of GPs screened their patients for retinopathy (85.6%), neuropathy (89.6%), nephropathy (88.8%) at some point in their follow up.

However a lower proportion of GPs screened their patients for diabetic foot (42.4%).At least an annual fundoscopic examination for retinopathy was carried out by majority of the GPs (75.2%), though only 20.8% examined the optic fundi after dilatation of pupils.

### Knowledge on diabetes

More than 95% of GPs accepted that a positive family history, obesity, sedentary life style, western food habits and gestational diabetes mellitus as risk factors for type 2 diabetes mellitus in Sri Lankans, despite not having documented evidence, while 94% felt that psychological stress was a risk factor for diabetes mellitus.

In this study 84.0% knew that microvascular complications of diabetes can be prevented by tight glycaemic control, while 71.2% were aware of the possibility of primary prevention of diabetes. Only 72.0% identified the cardiovascular diseases as the leading cause of death in those with diabetes. However only 50.4% knew the common association of retinopathy and nephropathy. Though nephropathy was identified as the cause of renal failure in diabetics by 87.2% of the GPs, only 36.8% knew that progression of nephropathy can be slowed down once established. When it comes to investigations only a minority knew the precise purpose of testing urine for microalbumin (39.2%) and measuring HbA1c levels (15.2%). GPs seem to be uncertain about how best to manage and follow up patients. Only a small number of GPs stated that blood pressure (58.4%), feet (30.4%) and weight (12.0%) as essential examinations that they perform in follow up visits.

Metformin was used as the drug of choice for patients who were overweight by 89.6% of the GPs and sulphonyureas were used by 88.0% for thin patients. Glitazones were used by 77.6% as the second line oral hypoglycaemic agent. In the management of female diabetic patient on glibenclamide desiring to be pregnant, 60.8% GPs admitted that they would change their therapy to insulin and 68.0% desired to take specialist opinion. There were 16.0% of the GPs who intended to continue glibenclamide and 23.2% preferred to stop the glibenclamide without starting on insulin or an alternative agent. Forty four percent of GPs used statins in patients with diabetes and ischaemic heart disease (IHD) irrespective of the cholesterol levels (table 2). Low dose aspirin was used by 32.8% of GPs in diabetic patients who were above 40 years of age while 87.2% used aspirin when patients had IHD and 80.8% when one or more IHD risk factors were present in addition to diabetes (Table 2). 87.2% use Angiotensin Converting Enzyme Inhibitors (ACEI) and Angiotensin Receptor Blockers (ATRB) as the first-line antihypertensives in their diabetic patients, while 54.4% used calcium channel blockers (CCB) as the second-line antihypertensive.

A high percentage of GPs advised their patients about diet control (96.8%) and exercise (84.0%) but only a few advised their patients to quit smoking (22.4%), foot care (29.6%) and retinopathy screening (15.2%).

The GPs' knowledge on current ADA cut-off values for metabolic and blood pressure control varied widely; correct values for triglycerides and HbA1c were identified respectively by 31.2% and 63.2% (Table 2). Only 28.8%% were aware of the cut-off values for LDL cholesterol for the commencement of statin therapy.

Knowledge of management of patients was also tested using case scenarios, results of which are shown in Table 3. Strict diet control (90.4%), quitting smoking(86.4%), starting statins(77.6%),starting anti-hypertensives (75.2%) and prescribing metformin(69.6%) were among the popular management steps of a diabetic who was 35 years old with 5 feet 6 inches in height, weighing 85 kg, blood pressure 140/100 mmHg, FPG 300 mg/dL, LDL cholesterol 200 mg/dL and smoking 10 cigarettes a day with a strong family history of heart attacks.

**Table 3:** The responses to the case scenario as preferred management options

Management option selected	Percentage of preferred responses
Strict diet control	90.4
Quit smoking	86.4
Regular exercise	81.6
Start on statins	77.6
Start on anti-hypertensives (ACE inhibitors/β blockers)	75.2
Start on Meformin	69.6
Weight reduction	66.9
Regular check for glycemic control	58.4
Start on aspirin	44.8
Examine eyes	21.6
Drug compliance	13.6
Examine foot	9.6

Only 20.8% and 11.2% of GPs knew that the UKPDS study and DCCT were done in patients with type-2 diabetes and type 1 diabetes respectively.

## Discussion and conclusions

In Sri Lanka, like in many other developing countries, remote rural locations lack government specialist medical clinics and those available in more urban areas are often overcrowded with patients. Thus GPs play a major role in management of patients with diabetes mellitus and providing international standards of care will result in improvement of clinical outcome.

It is encouraging to note that a majority of GPs screen for eye, neurological, renal and microvacular complications on newly diagnosed patients. However, only a minority do it with precise knowledge (e.g. low level of knowledge on the exact purpose of urine for microalbumin and HbA1c) and using the correct methods (as demonstrated by very low percentage dilating the fundi prior to ophthalmoscopy). Furthermore, an appreciable proportion of GPs were not aware of the diagnostic tests (e.g. need to use the glucose tolerance test) for the diabetes and the respective cut-off values based on current international guidelines (e.g. cut-off of 126 mg/dL for fasting plasma glucose).

A majority of GPs did not consider other complications like diabetic foot disease, dyslipidaemia, hypertension and obesity as important issues in patients, as demonstrated by the small number of GPs consider that routinely measuring blood pressure (58.4%), examining the feet for complications (30.4%) and checking weight (12.0%) as essential examinations that should be regularly done during follow up visits of diabetic patients. Similar studies performed in western countries have indicated better performance by GPs in recording blood pressure, ranging from 87% to 100% [12-14]. Although 42% screen for diabetic foot only 30% took time to offer adequate advice for the patients. The disparity is likely to be due to lack of time or lack of motivation. Having a standard printed set of patient information leaflets or trained health educationists may help to improve this situation. This discrepancy between knowledge and practice among GPs, is further demonstrated by the fact that although 96.8% advise about diet control and 84% about exercise but only 12% measure the weight and 58.4% do the blood pressure check-up during follow up.

Annual screening for lipids among Sri Lankan GPs was low (31.2%) when compared to 56% in United Kingdom [14] and 45% in USA [15]. However, Sri Lankan GPS often lack qualified support staff (e.g. qualified managers to manage the appointments, enter data, type letters, obtain reports from the laboratories etc.) and may deliberately avoid requesting for more effective tests (e.g. HbA1c) due to patients not being able to afford them.

The results of the case scenarios showed that the subjects correctly selected the class of oral hypoglycaemic agents and antihypertensive to suit the diabetic patient. However practices regarding use of aspirin and statins in diabetic patients were unsatisfactory. Most GP's use FBG and HbA1c to assess glyceamic control but some continue to rely on urine reducing substances. The awareness of Sri Lankan GPs on the current ADA target values for metabolic control with regard to blood glucose was inadequate and most lacked adequate knowledge on the management of diabetes in pregnancy (e.g. need to avoid sulphonylureas and start insulin).

Only a very few GPs were aware of important clinical trials, reflected by the lower level of awareness on landmark clinical trials on diabetes such as UKPDS (20.8%) and DCCT (11.2%) which was comparable to results from Pakistan [16]. This deficiency in knowledge of the recent advances in management protocols is most likely due to lack of continuous medical education among Sri Lankan GPs. This study identifies the fact that avenues to update their knowledge are meagre and it needs to be rectified by continuous medical education activities.

The study had several limitations. The response rate could have been improved in the future by increasing the coordination and introducing feedback to GPs. The study was not able to ensure whether some of the responders read textbooks or consulted their colleagues to answer the questionnaire. Though the responders were discouraged from looking up references, some might not have obeyed the instructions affecting data validity. Irrespective of this, there is an urgent necessity to develop education programmes to improve the knowledge of GPs, and subsequently audit their performance. Second we failed to explore the use of guidelines by the GPs. All currently available guidelines in Sri Lanka may not be appropriate for use by GPs in their busy clinics and we need to make necessary modifications to them to suit the context. Finally, members of the CCGP may not be representative of the complete population of GPs in the country. However, one could argue that since they are members of the only professional College dedicated to the specialty and they had considerable experience (mean duration of working as GPs for 28.7 years) their practice and knowledge ought to reflect those of the better GPs in the country.

Since this study very well depics the gap between the knowledge and practice there is an identified risk of management of some diabetes patients in Sri Lanka. Thus it is recommended to go ahead with outcome studies of patient management in units that are complying/not with guidelines.

In conclusion, the study found that there is much room for improvement in knowledge and practices related to diabetes among GPs. We recommend continuing medical education and regular training programs to update their knowledge in order to improve health outcomes in this group of patients. Further studies to investigate whether outcomes of diabetic patients (e.g. glycaemic control) relate to knowledge and practice among GPs are indicated.

## Abbreviations

GP: General practitioner; CCGP: Ceylon College of General Practitioners; FPG: Fasting Plasma Glucose; PPBS: Post Prandial Blood Sugar; UKPDS: United Kingdom Prospective Diabetes Study; DCCT: Diabetes Control and Complications Trial.

## Competing interests

'The authors declare that they have no competing interests.

## Authors' contributions

PK, GR, YS and MW carried out the study design, participated in data collection and drafted the manuscript. YS and JG participated in the design of the study and performed the statistical analysis. PK, DM, RS, MW, GR and PW conceived of the study, and participated in its design and coordination. All authors read and approved the final manuscript.
